# Dynamics of *Plasmodium falciparum* gametocyte carriage in pregnant women under intermittent preventive treatment with sulfadoxine–pyrimethamine in Benin

**DOI:** 10.1186/s12936-018-2498-8

**Published:** 2018-10-11

**Authors:** Sayeh Jafari-Guemouri, Jamila Dhiab, Achille Massougbodji, Philippe Deloron, Ndam N. Tuikue

**Affiliations:** 1 UMR 216 Mère et Enfant Face aux Infections Tropicales, Institut de Recherche pour le Développement (IRD), 4, Avenue de l’Observatoire, 75270 Paris, France; 2 Université Paris Descartes, Sorbonne Paris Cité, 75006 Paris, France; 3 Centre d’Etude et de Recherche pour le Paludisme associé à la Grossesse et à l’Enfance, Faculté des Sciences de Santé, Université d’Abomey-Calavi, 01 BP 188 Cotonou, Benin; 4 Noguchi Memorial Institute for Medical Research, University of Ghana, P.O. Box LG 581, Accra, Ghana

**Keywords:** Pregnant women, Malaria, *Plasmodium falciparum*, Intermittent preventive treatment, Gametocytes, Malaria transmission

## Abstract

**Background:**

In sub-Saharan Africa, malaria is a major cause of morbidity and mortality, in particular in children and pregnant women. During pregnancy, *Plasmodium falciparum* infected red blood cells expressing VAR2CSA are selected from circulation by selective cytoadherence to chondroitin sulfate proteoglycan receptors expressed in the placenta, leading to an increased susceptibility to malaria, long-lasting infections and poor pregnancy outcome. Partly because of these long-lasting infections, women were reported to have a higher density of gametocytes in their peripheral blood, and are considered as a potential reservoir for malaria transmission. To improve pregnancy outcome in areas of high malaria transmission, The WHO recommends intermittent preventive treatment with sulfadoxine/pyrimethamine (IPTp-SP) during antenatal care visits. The effect of IPTp-SP on gametocyte carriage in infected pregnant women was studied.

**Methods:**

The levels of transcription of three gametocytes stage-specific genes Pfs16 (expressed by sexually-committed ring stage parasites and fully matured gametocytes), Pfs25 (expressed by female mature gametocytes) and Pfs230 (expressed by male mature gametocytes) were assessed by real-time PCR in 50 *P. falciparum* infected women at early pregnancy (before implementation of IPTp-SP), and in 50 infected women at delivery. Sex ratios of male and female gametocytes were determined in these women to assess the effect of IPTp-SP on the gametocyte populations.

**Results:**

The data show that the three transcript types specific to Pfs16, Pfs25 and Pfs230 were detected in all samples, both at inclusion and delivery. Levels of Pfs25 and Pfs230 transcripts were higher at delivery than at inclusion (p = 0.042 and p = 0.003), while the opposite was observed for Pfs16 (p = 0.048). The ratio of male/female gametocyte transcript levels was higher at delivery than at inclusion (p = 0.018). Since a mixed gender late stage gametocyte culture was used as a positive control, male and female gametocytes could not be quantified in an absolute way in the samples. However, the amplification reliability of the Pfs25 and Pfs230 markers in the samples could be checked. A relative quantity of each type of Pfs transcript was, therefore, used to calculate the sex ratio proxy.

**Conclusion:**

This study demonstrates that IPTp-SP treatment contributes to modify the parasite populations’ structure during pregnancy. In line with previous studies, we suggest that the continued use of SP in pregnant women as IPTp, despite having a beneficial effect on the pregnancy outcome, could be a risk factor for increased transmission. This reinforces the need for an alternative to the SP drug for malaria prevention during pregnancy.

## Background

Malaria has a devastating impact in humans and is a major cause of worldwide morbidity and mortality. In sub-Saharan Africa, children and pregnant women are the two most vulnerable populations. Malaria has substantial risks for the pregnant woman (anaemia), her fetus (intra-uterine growth retardation), and the newborn child (low birth weight). Women in areas of high endemicity are more frequently infected with placental-type *Plasmodium falciparum* during their first and second pregnancies. This increased susceptibility at adulthood is due to the lack of specific immunity to the variant surface proteins that those parasites express on the surface of infected erythrocytes. In fact, the placenta that appears and develops as a new organ in the pregnant woman is a protected environment with new receptors to select parasites with uncommon cytoadherence properties throughout pregnancy [1, 2]. Because of this, an increased susceptibility to malaria and a prolonged infection due to placental sequestration have been documented during pregnancy [3, 4]. As part of the consequences of these long-lasting infections, women are expected to have a higher amount of gametocytes in their peripheral blood and are considered as a potential reservoir for malaria transmission [5].

These sexual and infective forms of the parasite can remain for several weeks in the woman’s peripheral circulation and can be transmitted to the mosquito during blood feeding [6, 7]. During the process of sexual reproduction of the parasite in the mosquito gut, new genotypes can emerge and be transmitted to a new human host when the mosquito becomes infectious and feeds again, 10–14 days after the initial blood meal. Studies in 2004 in Sudan have shown that pregnant women were more attractive to the mosquito and, therefore, possibly have disproportionate contribution to the infectious reservoir [8]. Pregnant women with long-lasting infection play an important role in malaria transmission to the mosquito. With this knowledge, the use of insecticides and bed nets among pregnant women in endemic areas deserve special valorization and attention.

The World Health Organization (WHO) recommends intermittent preventive treatment with sulfadoxine/pyrimethamine (IPTp-SP) during antenatal care visits for malaria prevention in pregnancy. IPTp-SP is effective on malaria and is effective in preventing the adverse consequences of malaria on maternal and fetal outcomes. This treatment is administrated from the second trimester of pregnancy at a 1-month interval [9]. The IPTp-SP treatment is effective only on asexual forms of the parasite, but not on gametocytes.

It has been shown that IPT favours gametocyte generation in children with acute uncomplicated malaria [10]. Furthermore, pregnant women receiving IPTp-SP have higher incidence of gametocytaemia compared to non-treated pregnant women [11, 12]. SP can cause the release of sequestered gametocytes into the peripheral blood that may persist for over a month [13–16]. A study in 1990 suggested that some asexual forms that are not eliminated by SP go through sexual differentiation leading to the presence of gametocytes in the blood [17]. Thus, while the use of IPTp-SP on one hand may help to reduce the parasitaemia of asexual forms in peripheral blood, it may increase the gametocytaemia on the other hand. Even though the sexual forms of *P. falciparum* are not responsible for clinical symptoms in infected pregnant women, their major role in malaria transmission justifies investigating the effect of IPTp-SP on gametocytes density in infected pregnant women.

The infection to the mosquito requires the mating of two gametes of opposite sex. Therefore, the proportion of male gametocytes in the human blood is a very important parameter to evaluate the infectivity of individuals to mosquitoes and to investigate factors affecting malaria transmission at population level [18]. The number of male gametocytes better determines infection success than that of female gametocytes [19]. A higher infectivity of gametocytes has been observed in the case of higher sex-ratios [20]. Among different clones from a single isolate adapted to in vitro culture, those with a higher male sex ratio were more infectious to mosquitoes [21]. Thus, the densities of female and male gametocytes in the blood, and their sex ratio influence infectivity to mosquitoes, and so, onwards transmission to new hosts.

The primary objective of this study was to assess the effect of IPTp-SP on gametocyte carriage in infected pregnant women. Gametocyte specific markers were used to compare gametocyte prevalence in malaria infected women at early pregnancy (before the implementation of IPTp-SP), and at delivery (after women have received 2 doses of SP). The secondary objective was to compare the sex ratio bias of parasite gametocytes in infected pregnant women at early pregnancy and at delivery to assess the effect of IPTp-SP on the gametocyte populations. Using sensitive molecular methods to detect transcripts of genes that are specifically expressed by male or female gametocytes under field conditions can be informative in monitoring the risk of transmission from humans to mosquitoes. Accurate quantification of the densities and ratio of female and male gametocytes in parasite carriers in the field will increase understanding of *P. falciparum* transmission and improve the evaluation of transmission blocking interventions.

## Methods

### Study population and samples collection

Samples were collected from pregnant women during the STOPPAM study conducted between 2008 and 2010 [22] in health centres of southern Benin where transmission of *P. falciparum* malaria is hyper-endemic with an entomological inoculation rate ranging from 35 to 60 infective bites per person and per year [23]. *Plasmodium falciparum* infection was tested by a rapid diagnostic test Parascreen™ (Zephyr Biomedicals Goa, India) and was confirmed by microscopy among pregnant women who presented to the health centres for their inclusion in the study at their first antenatal visit and during successive visits until delivery. Women were enrolled after obtaining signed informed consent, and venous blood was collected. Eight milliliters of venous blood were collected from women with plasmodial infection, in vacutainers with citrate phosphate dextrose adenine anticoagulant. Two hundred microlitre of erythrocyte pellets were homogenized in 10 volumes of TRIzol reagent (Invitrogen, Life Technologies, France) and stored at − 80 °C until RNA extraction. Detailed characteristics of the study sites have been described [24]. One hundred women with available clinical data throughout the follow-up and having parasitized samples (RDT+) (Rapid Diagnostic Test) at first antenatal visit (n = 50) or at delivery (n = 50) were included. Table 1 shows parameters for the two groups of women. All studies were approved by the Ethics Committee of the Faculty of Health Sciences, University of Abomey-Calavi (Benin).

**Table 1:** Characteristics of the two groups of 50 infected women by *Plasmodium falciparum* selected at inclusion and at delivery

Characteristics	Inclusion	Delivery
Parasitaemia (median [IQR]) (parasites/µl blood)	275 (126–563)	456 (0–2121)
Primigravidae (%)	19/47 (40%)	8/50 (16%)
Gestational age (median [IQR]) (weeks)	16.29 (14.35–17.55)	38.65 (36.95–41.05)
Maternal haemoglobin (median [IQR]) (g/dl)	9.8 (9.3–10.8)	10.9 (9.8–11.7)
Maternal age (median [IQR]) (years)	22 (19–28)	25 (20.5–30)

The parasitaemia presented are those deduced from microscopy reading. Infections detected by PCR have not been quantified

### RNA extraction and cDNA synthesis

Infected erythrocyte pellets collected from pregnant women were conserved in TRIzol reagent and stored at − 80 °C. After homogenization, 200 µl of chloroform (Merck, Germany) were added to 900 µl of sample, agitated for 15 min followed by 5 min incubation at room temperature. The samples were centrifuged for 30 min at 14,000 rpm at 4 °C, and conserved in the ice afterward. The aqueous phase containing the total RNA was separated from the DNA, and transferred to a fresh tube and an equal volume of isopropanol (4 °C) was added. The samples were incubated overnight at − 20 °C (RNA precipitation) then centrifuged for 30 min at 14,000 rpm at 4 °C. The following steps were performed on ice until reverse transcription. The supernatants were removed and the pellets containing RNA were re-suspended in 800 µl of ethanol 75%, vortexed, and centrifuged for 10 min at 14,000 rpm at 4 °C. The supernatant were removed and the pellets were dried at room temperature and resuspended in 10 µl of sterile RNase-free water. To avoid contamination with DNA, the samples were treated with DNase I (Invitrogen, France) for 30 min at room temperature. The absence of gDNA in all RNA samples was confirmed by no parasite DNA amplification after 40 cycles of real-time PCR performed with seryl-tRNA synthetase (P90) *P. falciparum*-specific primers, using a Rotorgene 6000 thermal cycler system (Corbett Research). P90 is a housekeeping gene expressed in all asexual and sexual form of the parasite) [2].

### Reverse transcription and quantification of specific gametocyte genes by real time PCR

The reverse transcription of DNA-free RNA was carried out using the Thermoscript Reverse Transcription System (Invitrogen, France) according to the manufacturer recommendations. This is a 2-step reaction. Briefly, a mix of random hexamer primers, Oligo dT, dNTP and RNA (Mix 1) in a total volume of 8 µl went through heating at 65 °C for 5 min. We added 8 µl of Mix 2 containing Buffer, DTT, RNase out, DEPC water and Thermoscript. This step of the reverse transcription was performed in a total volume of 16 µl as follows: 25° for 10 min, 50 °C for 25 min and 85 °C for 5 min. The cDNA was conserved at − 20 °C. The levels of transcription for three stage-specific genes Pfs16, Pfs25 and Pfs230 have been assessed in previously developed real-time PCR protocols [25, 26]. Pfs16 is known as a marker for detection of sexually committed ring stage parasites and fully matured gametocytes [27, 28]. Previous studies have also shown that Pfs16 plays an important role in the process of gametocytes maturation [29]. It is an early marker of the development of mature gametocytes in culture, and is therefore a potential target for new anti-malarial drugs [25]. Pfs25 and Pfs230 are used for the detection of stage V female mature gametocytes and male mature gametocytes, respectively [26]. Pfs25 is currently used to quantify gametocytes from field isolates [30, 31].

The transcript levels of Pfs25, Pfs16 and Pfs230 genes were quantified in a real time quantitative PCR reaction containing 1 µl of cDNA, 6 µl of 2× SensiFast SYBR No-ROX (Bioline), and 1 µM of specific primer pairs (IDT) and sterile water qsp 12 µl. The primers’ sequences are as follows: Pfs 25 Forward primer (5′–3′) TCTTTTCCTTTTCATTCAACTTAGCA, Pfs 25 Reverse primer (5′–3′) CCACTCATCTGAATTAAAAATCCTCTT; Pfs16 Forward primer (5′–3′) TGCAAACCCCAGGAAGTTCT, Pfs16 Reverse primer (5′–3′) AAAGACCTTGAGATAGTCCACCTAGAT [25]; Pfs230 Forward primer (5′–3′) CCCAACTAATCGAAGGGATGAA; Pfs230 Reverse primer (5′–3′) AGTACGTTTAGGAGCATTTTTTGGTAA [26]. P90Forward primer (5′–3′) AAGTAGCAGGTCATCGTGGTT; P90 Reverse primer (5′–3′) TTCGGCACATTCTTCCATAA [2].

Multiple independent quantitative PCR with four markers P90, Pfs16, Pfs25 and Pfs230 were performed in a Rotorgen 6000 with the following cycle profile: 95 °C for 10 min, followed by 40 cycles amplification with 5 s denaturation at 95 °C, 15 s annealing at 59 °C and 20 s elongation at 72 °C. A Cycle Threshold (CT) value was assessed for Pfs16, Pfs25 and Pfs230 of each sample. If the CT value exceeded 35, the transcript was estimated as non-quantified. No template controls and 3D7 gDNA were used as calibrator for validation in every run. The cDNA from a mixed gender late stage gametocyte culture was used as a positive control for all samples (INSERM U1016, Institut Cochin, Paris, France). The melting curve analysis was done for the four transcripts (Pfs16, Pfs25, Pfs230 and P90) in all samples to ensure the amplification specificity. The PCR efficiency was assessed by a standard curve for each gene with a serial dilution of a 3D7 genomic DNA and validated on serial dilution of cDNA from the gametocyte culture. Seryl-tRNA synthetase (primer pair P90) was used as endogenous controls as it is expressed in all parasite stages [2]. Non-template controls and the 3D7 gDNA (calibrator) were performed for validation on every run.

### Data analysis

For each sample and each of the three gametocyte markers (Pf16, Pfs25 and Pfs230), the relative abundance was estimated relative to that of the *P. falciparum* seryl-tRNA synthetase gene from the same sample as delta CT values (ΔCT). ∆CT from qPCR reactions were calculated as the difference between the CT of the studied gene and the endogenous gene seryl-tRNA synthetase. The ratio of male/female gametocytes transcripts level at inclusion and delivery was calculated based on the relative copy numbers of Pfs25 and Pfs230 expressed as TU (transcript units). Samples corresponding to transcript levels with ΔCT value > 5 were assigned to ΔCT = 5 and therefore, TU was equal to 1 [32]. Unpaired t-test was used for comparisons between groups. All calculations were performed and data were analyzed with GraphPad Prism6 software (RITME Scientific Solutions, Paris, France). p-values < 0.05 were considered statistically significant. All p-values are indicated within the figures or the text as appropriate.

## Results

Characteristics of the two groups of 50 infected women selected at inclusion and 50 infected women selected at delivery are summarized in Table 1. The median for the parasitaemia, the proportion of primigravidae, the gestational age, the maternal haemoglobin and the maternal age of those infected women are shown. 18s-qPCR confirmed the presence of P. falciparum DNA in all selected samples.

**Fig. 1:**
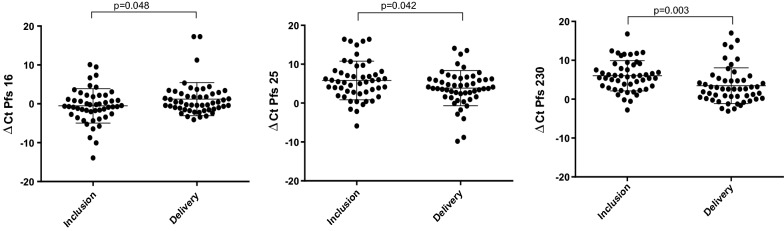
Level of Pfs16, Pfs25 and Pfs230 transcripts at inclusion and delivery in pregnant women infected by *P. falciparum* in Benin. Time points are represented on the horizontal axis for inclusion and delivery groups. Each point indicates ΔCT value for one pregnant woman. Medians, 25% and 75% percentiles (error bars) are represented for each marker with horizontal bars. Statistical significances are labeled above individual charts

The qPCR data show that the three transcript types specific to Pfs16, Pfs25 and Pfs230 were detected in all samples, both at inclusion and delivery. The levels of Pfs25 and Pfs230 transcripts expressed as ∆CT were higher at delivery compared to inclusion (p = 0.042 and p = 0.003). However, the Pfs16 ∆CT was lower at delivery compared to inclusion (p = 0.048) (Fig. 1).

**Fig. 2:**
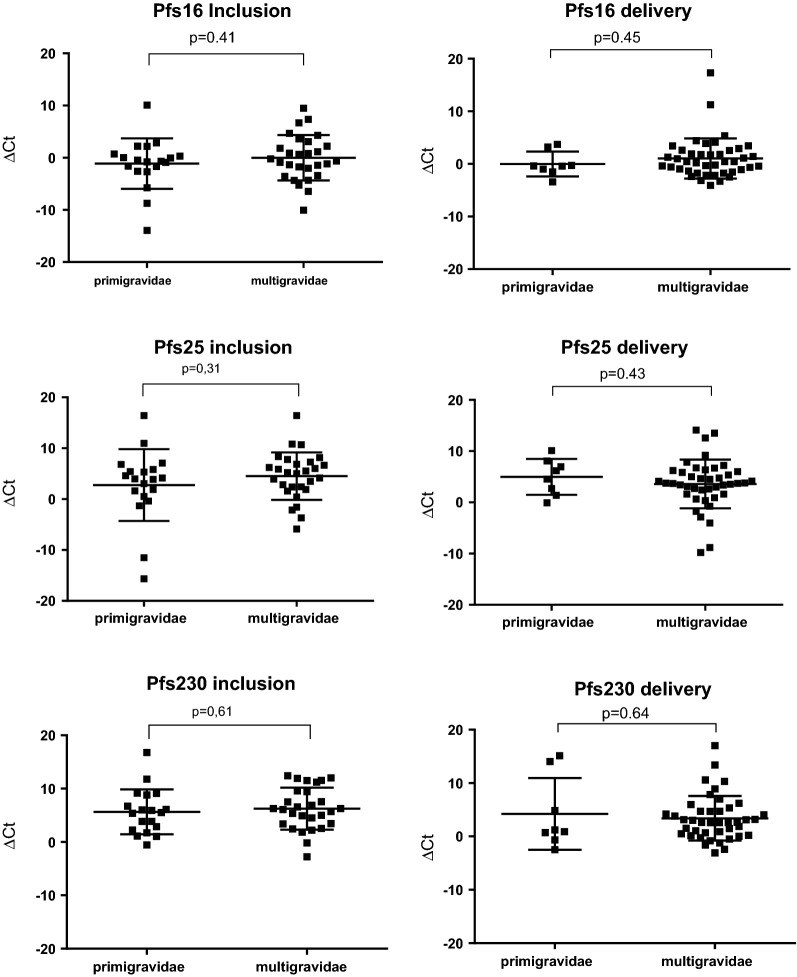
Level of Pfs16, Pfs25 and Pfs230 transcripts at inclusion and delivery in primigravidae and multigravidae women infected by *Plasmodium falciparum* in Benin. Each point indicates ΔCT value for one pregnant woman. Medians, 25% and 75% percentiles (error bars) are represented for each marker with horizontal bars. Statistical significances are labeled above individual charts

**Fig. 3:**
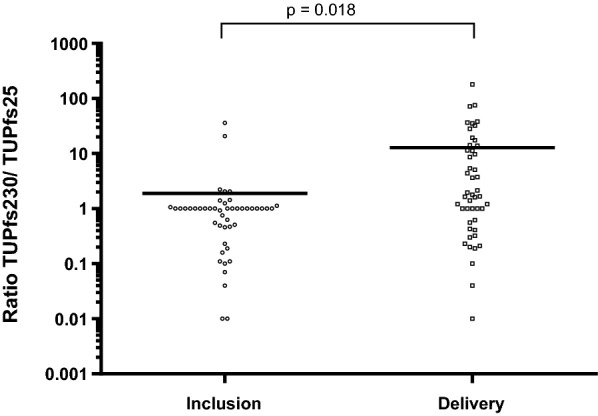
Ratio of male/female gametocyte transcript levels at inclusion and delivery in infected pregnant women in Benin. *TU* transcript unit: low abundant transcript with ΔCT values > 5 are assigned to ΔCT = 5. For the corresponding samples, TU = 1 (Lavstsen et al. [32]). For the TU values, logarithmic scale base 10 is used

The level of Pfs16, Pfs25, and Pfs230 transcripts between primigravidae and multigravidae was compared at inclusion and delivery and found no significant difference due to parity at any of the time points. The distribution of ∆CT values for the three *P. falciparum* transcripts at inclusion and delivery are shown in Fig. 2. The ratio of male/female gametocyte transcript levels was however higher in the delivery group compared to the inclusion group (p = 0.018) (Fig. 3).

## Discussion

This study is the first attempt at profiling *P. falciparum* gametocytaemia in a cohort of malaria exposed pregnant women under IPTp-SP, and also one of the few studies examining gametocytaemia in the context of pregnancy [5, 11, 33]. Based on the WHO recommendation in 2004, Benin like other countries in sub-Saharan Africa had adopted IPTp with 2 or 3 doses of sulfadoxine–pyrimethamine given from second trimester of pregnancy at 1-month interval. Several studies have shown that the administration of treatment doses of SP can lead to maintenance of a chronic asymptomatic parasitaemia, which to some extent can contribute to poor pregnancy outcomes (including low birth weight, maternal anaemia) [34]. Recent studies from East Africa have reported frequent cases with consequences of SP-resistance on pregnancy outcomes [35, 36].

Despite the decline in efficacy, IPTp-SP remains active to reduce poor pregnancy outcome in Africa and is the only drug that can be safely administered in pregnant women. The low efficacy to clear parasites explains the many recrudescences observed in the cohort study on which the current work was designed [24], but it could also contribute to the formation of gametocytes, making pregnant women a non-negligible reservoir for transmission. It has been suggested that IPTp-SP releases sequestered gametocytes into the peripheral blood that may persist in the blood for over a month [13, 14, 16]. Some asexual forms that are not eliminated by SP go through sexual differentiation [17]. Thus, the use of IPTp-SP may increase gametocytaemia in the peripheral blood of the pregnant woman and, therefore, impact the level of transmission.

Very few studies investigated the dynamic of infecting parasites in pregnant women as well as the mechanism of transmission of malaria by gametocytes. As pregnant women are at higher risk of malaria compared to non-pregnant adults in the community, infected pregnant woman may represent an important reservoir of gametocytes and it is, therefore, of interest to analyze the dynamic of sexual forms of *P. falciparum* in the pregnant woman, and the impact of IPTp-SP on this phenomenon. A previous study has reported that SP increased gametocytaemia in children living in Mali [14]. The present study characterizes gametocyte populations with several markers early in pregnancy and at delivery in two selective cross-sections of pregnant women under IPTp-SP. The level of expression of three specific transcripts markers (Pfs16, Pfs25 and Pfs230) has been assessed to identify and evaluate early and late-stages gametocytes in peripheral blood. Gametocytaemia was observed in all the women in the two selective cross sections. However differences between infected primigravidae and multigravidae were not shown, neither at inclusion nor at delivery indicating that infecting parasites behave similarly in these groups for this factor. Using sensitive molecular methods to detect gametocytes under field conditions can be informative in monitoring the risk of malaria parasites transmission from human hosts to propagating mosquito vectors. Accurate quantification of the densities and ratio of female and male gametocytes in parasite carriers in the field will increase our understanding of *P. falciparum* transmission and improve the evaluation of transmission blocking interventions.

Previous studies have shown that Pfs16 plays an important role in the process of gametocyte maturation as it is present during the entire gametocyte maturation from stage I to V [37, 38]. In this study, the mean level of Pfs16 transcripts was higher at inclusion than at delivery. This is because Pfs16 targets different stages of the parasites which are fully mature gametocytes and sexually committed ring stage parasites [27, 28]. At inclusion, when women have not received any dose of IPTp yet, Pfs16 marker identifies both asexual and sexual forms in the blood circulation. After two doses of IPTp-SP, a decline in parasite densities has been shown in the whole cohort that was previously published [24], but because of re-infections and recrudescences that occurred later, parasite populations’ structure was affected and the balance between asexual and sexual forms was modified. It is therefore very likely that IPTp-SP treatment can strongly contribute to the modification of the parasite populations’ structure between the two time points of the follow-up and that the whole range of the parasite stages targeted by the Psf16 marker is not yet reconstituted on the parasite populations seen at the delivery, hence the decrease the PfS16 transcript level observed.

The levels of Pfs25 and Pfs230 transcripts indicate that both male and female gametocytes were significantly higher at delivery in infected pregnant women having received IPTp-SP compared to those seen at inclusion prior to SP treatment. These data also clearly showed for the first time a bias in the sex ratio of late stage gametocytes after IPTp-SP uptake, in favour of more male gametocytes which may have implication in increasing the infectivity of these forms, as suggested by a previous report on the infectivity of *P. falciparum* to *Anopheles gambiae* [20, 39].

Regarding the influence of sex ratio on infectivity, previous works have shown that there is a complex relationship between gametocyte density and mosquito infection. A study in 2009 has shown that while increasingly male sex ratios do give higher transmission success at low gametocyte densities, they reduce success at higher densities [39]. A study in 2018 has shown that transmission from low gametocyte densities may be impeded by a lack of male parasites while the proportion of mosquitoes infected is primarily determined by the density of female gametocytes. This work highlights the importance of measuring both female and male gametocyte density when estimating the human reservoir of infection [40].

A study by Hanson in 1935 [19] had already postulated that the number of males, rather than female gametocytes, determines infection success. Moreover, in vitro transmission studies using different clones of *P. falciparum* from a single isolate showed that the clones with a higher male sex ratio were more infectious to mosquitoes [21]. Other in vitro studies of mosquito infection rates found no effect of the gametocyte population structure, including sex ratio, on infection success [41]. A cross-sectional study assessing the effect of the sex ratio on the infectiousness of natural *P. falciparum* infections in man revealed the same tendency for highest transmission success to be associated with a higher proportion of male gametocytes [20].

The data suggest that IPT increases the level of both males and females gametocytes, with a balance leaning toward the preferential production of male gametocytes. This observation questions the mechanism behind this bias, and the risk that IPTp-SP may ultimately increase the reservoir of transmission in pregnant women. To confirm the effect of IPTp-SP on the evolution of gametocytes from early to mature stage and on sex ratio, a larger sampling size is required; an in vitro study of the dynamic of the male and female gametocytes maturation under SP pressure will be relevant. Moreover, since a mixed gender late stage gametocyte culture was used as a positive control, male and female gametocytes were not quantified in an absolute way in studied samples. However, this has allowed us to ensure the amplification reliability of the Pfs25 and Pfs230 markers. The ability to generate pure cultures of male or female gametocyte remains technically difficult to achieve to date for a more precise quantification of each gender. In this study, a relative quantity of each type of Pfs transcript based on that of a parasite housekeeping gene was used to calculate the sex ratio proxy.

The intended purpose of IPTp-SP is to reduce asexual stages parasite loads to improve pregnancy outcome. The data from this study indicate an increase in gametocytaemia at delivery in infected pregnant women who are under IPTp-SP. A study conducted among pregnant women at the beginning of the implementation of IPTp-SP in Gabon in 2005 and 6 years later had already pointed to an increase of sub-microscopic gametocyte carriage [33]. While these observations suggest that IPTp-SP reduces the asexual parasitaemia, the current data conversely suggest an increase in gametocytaemia among infected women. In line with previous studies [11, 42–44], the continued use of SP in pregnant women as IPTp, although still having a beneficial effect on the pregnancy outcome, may be a factor favouring transmission. This reinforces the need to find an alternative to the SP drug.

## Perspectives

Whether IPTp-SP modifies infectiousness of the gametocytes and/or the level of transmission via the mosquito is unknown. In a previous study, human infectious reservoir has been determined by studying adults and submicroscopic gametocyte carriers in an endemic area in North Senegal [45]. A similar study can be carried out to compare the level of gametocytes infectiousness before and after IPTp-SP treatment in a group of pregnant women, by feeding mosquitoes from infected pregnant women blood collected before and after IPT treatment. The level of Pfs25 and Pfs230 transcripts in pregnant women before and after IPTp-SP treatment will confirm its effect in gametocyte infectivity from human to mosquitoes, and inform us about the role of the IPT in the transmission of malaria. This should be taken into consideration in malaria control and elimination strategies.
